# X‐Ray Crystallography and Free Energy Calculations Reveal the Binding Mechanism of A_2A_ Adenosine Receptor Antagonists†

**DOI:** 10.1002/anie.202003788

**Published:** 2020-07-22

**Authors:** Willem Jespers, Grégory Verdon, Jhonny Azuaje, Maria Majellaro, Henrik Keränen, Xerardo García‐Mera, Miles Congreve, Francesca Deflorian, Chris de Graaf, Andrei Zhukov, Andrew S. Doré, Jonathan S. Mason, Johan Åqvist, Robert M. Cooke, Eddy Sotelo, Hugo Gutiérrez‐de‐Terán

**Affiliations:** ^1^ Department of Cell and Molecular Biology Uppsala University, BMC, Biomedical Center Box 596 Uppsala Sweden; ^2^ Sosei Heptares Steinmetz Granta Park, Great Abington Cambridge CB21 6DG UK; ^3^ Departament of Organic Chemistry Faculty of Farmacy Universidade de Santiago de Compostela Spain; ^4^ Centro Singular de Investigación en Química Biolóxica y Materiais Moleculares (CIQUS) Universidade de Santiago de Compostela Spain; ^5^ Present address: H. Lundbeck A/S Ottiliavej 9 2500 Valby Denmark

**Keywords:** adenosine receptors, biophysical mapping (BPM), free energy perturbation (FEP), G protein-coupled receptor (GPCR)

## Abstract

We present a robust protocol based on iterations of free energy perturbation (FEP) calculations, chemical synthesis, biophysical mapping and X‐ray crystallography to reveal the binding mode of an antagonist series to the A_2A_ adenosine receptor (AR). Eight A_2A_AR binding site mutations from biophysical mapping experiments were initially analyzed with sidechain FEP simulations, performed on alternate binding modes. The results distinctively supported one binding mode, which was subsequently used to design new chromone derivatives. Their affinities for the A_2A_AR were experimentally determined and investigated through a cycle of ligand‐FEP calculations, validating the binding orientation of the different chemical substituents proposed. Subsequent X‐ray crystallography of the A_2A_AR with a low and a high affinity chromone derivative confirmed the predicted binding orientation. The new molecules and structures here reported were driven by free energy calculations, and provide new insights on antagonist binding to the A_2A_AR, an emerging target in immuno‐oncology.

## Graphical abstract

**In molecular design**, structural, pharmacological and chemical information can be interconnected by computational estimations of binding free energies. Using a combined FEP approach to examine both mutagenesis and ligand SAR, we designed new analogues of the A_2A_AR antagonist series of chromones. Subsequent crystal structures supported the rational design of these compounds, linking the structural and energetic understanding on ligand binding.

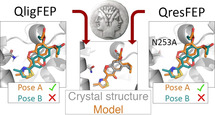

## Introduction

Computational estimation of shifts in binding free energy, associated with ligand modifications or point mutations in the receptor macromolecule, can provide the missing link between the structure of a protein‐ligand complex and a panel of experimental binding affinities. Rigorous free energy perturbation (FEP) methods have been used for decades to understand the structure‐affinity relationships (SAR) around a given chemical scaffold, and recent advances now allow the use of this technique routinely in ligand design projects.^1^ The same methodology can be used to analyze this problem from a complementary perspective, that is to estimate the gain or loss in binding free energy from site‐directed mutagenesis (SDM) data. The idea of in silico mutagenesis, initially introduced almost three decades ago by Kollman to study the binding and catalysis of subtilisin,^2^ was recently implemented in computational pipelines that pursue a systematic characterization of the effect on point mutations on for example, ligand‐binding or protein stability.^3^, ^4^, ^5^ The combination of both ligand and residue FEP simulations can provide a full energetic landscape of the molecular interactions governing protein‐ligand binding, which underlies the design of two complementary protocols in our lab, namely QligFEP^6^ and QresFEP,^3^ integrated in the molecular dynamics (MD) software package Q.^7^, ^8^

One area where this approach is particularly promising is the design of ligands for G‐protein‐coupled receptors (GPCRs), a superfamily of seven‐transmembrane (7TM) cellular receptors^9^ that mediate the therapeutic effects of about 30 % of all marketed drugs.^10^ There is a large amount of SAR and SDM data available for these receptors, which can be combined with the increasing growth of structural knowledge of many GPCR targets. The first integrated approach of ligand and residue FEP simulations was published by Boukharta et al. to characterize antagonist binding to the Y_1_ neuropeptide receptor,^11^ which we later expanded to other GPCR families, including the related neuropeptide receptor Y_2_,^12^ the orphan receptor GPR139^13^ and several members of the family of adenosine receptors.^14^, ^15^, ^16^ Among the latter, the adenosine A_2A_ receptor (A_2A_AR) was one of the first GPCRs to be crystallized^17^ and today stands out as one of the better characterized GPCRs from a structural perspective. Several structures of the inactive and active forms of the receptor have been solved within the last decade, and the integration of the available experimental data has strongly aided ligand design programs for this receptor.^18^, ^19^

Many A_2A_AR antagonists have been developed targeting a number of pathologies,^20^, ^21^, ^22^ including recent clinical candidates in immuno‐oncology. A number of these antagonists have been co‐crystallized with the A_2A_AR, providing unique structural information which, in combination with the extensive SDM data available,^23^ allow envisaging ligand binding mechanisms and structure‐based drug design (SBDD) programs of antagonist molecules.^18^ However, suboptimal properties of traditional scaffolds, such as poor pharmacokinetics and low selectivity profiles, motivate the search of novel chemical entities as A_2A_AR antagonists,^24^ frequently through high throughput screening (HTS) campaigns. In these cases, it is not common to obtain a crystal structure of the receptor‐ligand complex, which can hamper further hit to lead optimization. Instead, approximate binding modes are often inferred from the experimental data extracted from SAR of ligand series and SDM data,^23^ which can be complemented by computational models of the protein‐ligand complex.^25^

**Figure 1:**
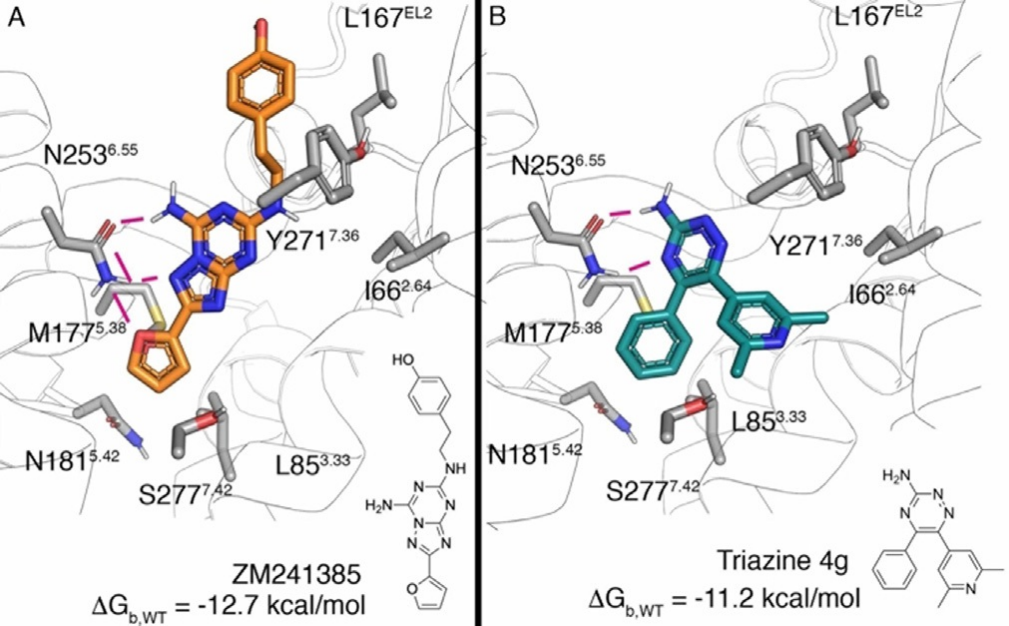
Binding mode and chemical structures of antagonists **ZM241385** (A, crystal structure 4EIY^26^) and triazine **4 b** (B). The experimental pose of the triazine (cyan) was superimposed on the same crystal structure of the receptor shown in panel A (ribbons). Both compounds had been characterized by BPM (residues labelled and depicted in gray sticks). Receptor‐ligand hydrogen bonds are depicted as magenta lines.

**Figure 2:**
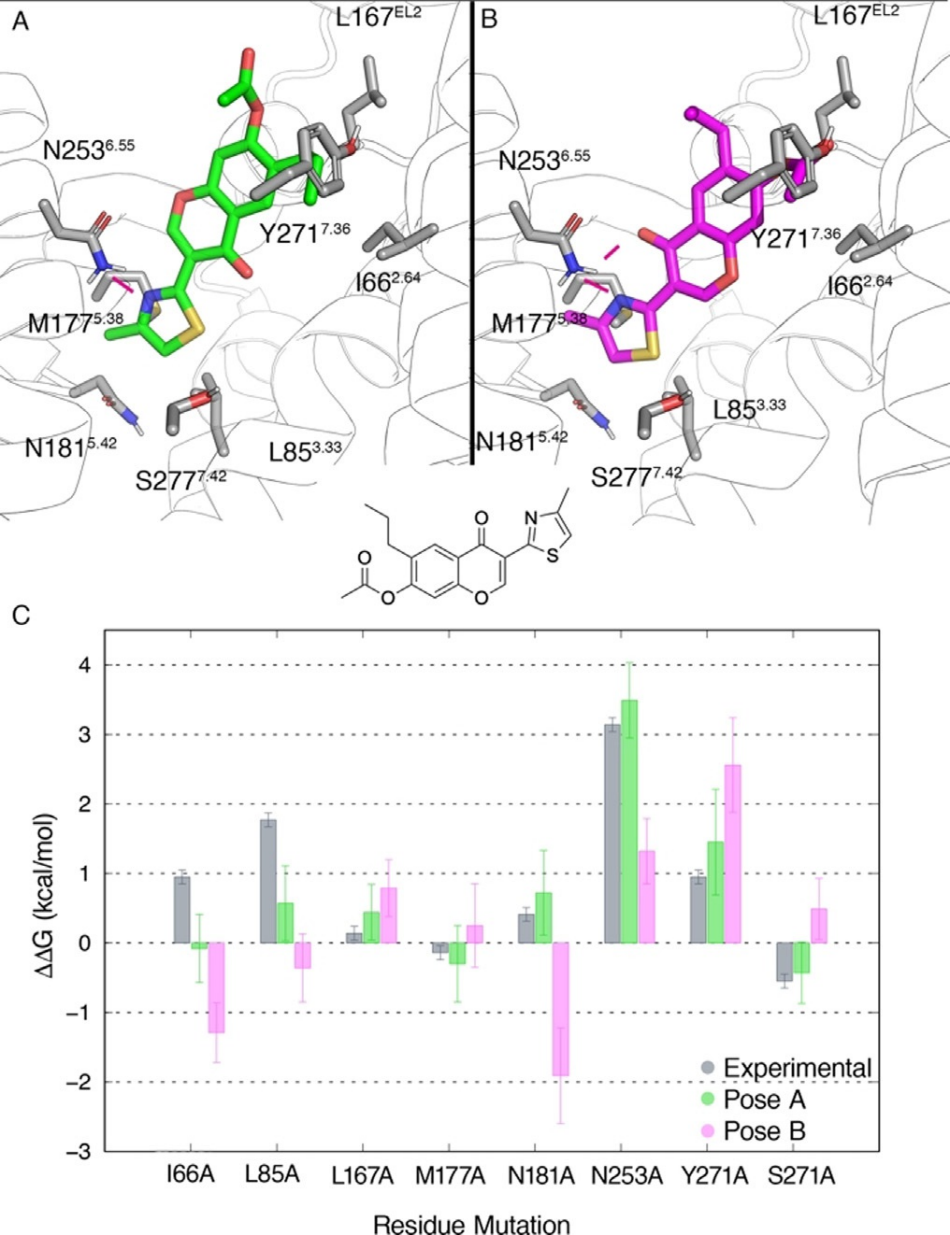
Putative binding modes A (green) and B (magenta) of **Chromone 14** to the A_2A_AR (H‐bonds in magenta). C) Experimental and calculated changes in binding free energies for each mutation in the BPM. The error bars correspond to the s.e.m. of the replica calculations for the calculated values, or are adjusted to the reported value of 0.1 p*K*_D_ unit in the case of experimental data.^27^

Biophysical Mapping (BPM), is an integrated approach that has been used with success in antagonist design programs on the A_2A_AR and other GPCRs.^27^, ^28^, ^29^, ^30^ Here, the binding affinity of a ligand series is evaluated via surface plasmon resonance (SPR) on a panel of mutant receptors, each bearing a single‐point mutation within the putative binding site.^27^ The resulting matrix of binding affinity shifts from wild type (WT) affinities combined with SAR data and mapped to a receptor‐ligand model provide further insights in the determinants of binding of the scaffold. The first application of this technique was based around the co‐crystallized A_2A_AR antagonist **ZM241385** in combination with 8 receptor mutants (see Figure 1 A).^22^ These mutations involve residues in direct contact with the ligand, such as N253^6.55^, L85^3.33^, M177^5.38^, N181^5.42^ and I66^2.64^ (Ballesteros Weinstein numbering^31^ in superscript), as well as residues not directly in contact with the ligand, namely S277^7.42^, Y271^7.36^ and L167^EL2^ (see Figure 1). This approach was extended for 1,2,4‐triazines as A_2A_AR antagonists,^32^ and the binding mode was later confirmed by X‐ray crystallography (see Figure 1 B).^29^ In the same HTS campaign, a series of chromones were identified as a novel family of A_2A_AR antagonists,^32^ and consecutively optimized to yield the potent and selective **Chromone 14** (see Figure 2).^30^ At that point, the lead‐optimization program was successful in improving the affinity of the initial HTS hit, while not focusing on pharmacokinetic optimization (i.e., the most potent compound **Chromone 14** contains a metabolically unstable ester group). Interestingly, this structure‐based optimization was guided by the interpretation of the BPM data and a computational model of the complex generated by docking. This led to the proposal of two putative binding modes compatible with the BPM data. Even if the SAR of the generated series seemed to favor one of them, the question remained open due to the lack of an X‐ray structure with any of these compounds in the original study.^30^, ^32^

Here, we initially examine the SPR data available for these A_2A_AR antagonist families through a recently developed in silico mutagenesis tool based on free energy perturbation (FEP) simulations.^11^, ^14^, ^15^ The results pointed to a unified binding mode of the chromone series, which is here used as a basis for the design, synthesis and pharmacological evaluation of an extended series of compounds aimed to further explain the underlying SAR of chromones as A_2A_AR antagonists. Finally, experimental structures of two chromone‐A_2A_AR complexes were solved which confirmed the binding mode hypothesis from the computational studies.

## Results and Discussion

### Free Energy Perturbation Calculations on Existing BPM Data

The BPM data obtained for the three chemotypes of A_2A_AR antagonists were collected from reference 27, and relative binding free energy changes between mutant and WT receptor were calculated from *K*_i_ values (see Table 1, ${\Delta \Delta G}_{bind}^{exp}$ ). Thereafter, relative binding free energies for each ligand were calculated based on 3D models of the receptor‐ligand complexes (${\Delta \Delta G}_{bind}^{calc}$ , Table 1). In the case of **ZM241385,** the model was directly extracted from the high‐resolution structure with the A_2A_AR (see Figure 1), and the curated receptor model (see Supporting Information, Methods) was used throughout this work. The results show excellent agreement with the experimental data, with a mean absolute error (MAE) of 0.41 kcal mol^−1^ and a correlation coefficient of R^2^=0.94 (Table 1). Next, the same set of BPM mutations was analyzed for triazine **4 g**. In this case, the starting configuration of the complex was obtained by aligning the triazine **4 g**‐A_2A_AR complex (3UZA) to the curated model described before, retaining only the coordinates of the ligand (Figure 1 B).^33^ The calculated free energies (${\Delta \Delta G}_{bind}^{calc}$ , Table 1) were in general good agreement with the experimental data (MAE=0.94 kcal mol^−1^; R^2^=0.66). In contrast to the previous case, two mutations (Met177^5.38^ and Asn181^5.42^) show qualitative discrepancy with the experimental data for this ligand. Subsequent analysis of the MD trajectories suggests that this is likely due to a suboptimal representation of the (water mediated) H‐bond network upon mutation, which has previously been recognized as a challenging factor for this particular scaffold.^34^, ^35^

**Table 1:** Comparison between experimental and calculated relative binding free energies ($\Delta \Delta G_{bind}$ in kcal mol^−1^) for A_2A_AR mutants.

Mutant^[a]^	ZM241385		Triazine **4 g**	
	${\Delta \Delta G}_{bind}^{exp}$ ^[b]^	${\Delta \Delta G}_{bind}^{calc}$	${\Delta \Delta G}_{bind}^{exp}$ ^[b]^	${\Delta \Delta G}_{bind}^{calc}$
I66A^2.64^	0.14	0.83±0.34	0.41	1.94±0.34
L85A^3.33^	2.45	3.30±0.41	1.09	1.65±0.37
L167A^5.28^	0.00	0.60±0.31	−0.14	−0.39±0.36
M177A^5.38^	0.14	−0.09±0.44	−0.27	1.66±0.49
N181A^5.42^	1.23	1.47±0.57	0.82	−0.63±0.55
N253A^6.55^	≥5.86^[c]^	5.81±0.57	≥4.36^[c]^	5.64±0.56
Y271A^7.36^	1.09	0.84±0.74	0.41	−0.1±0.68

[a] Data for the mutant receptor constructs reported in reference 27. [b] Experimental relative binding free energies were calculated from *K*_D_ values as ${\Delta \Delta G}_{bind}^{exp}=RTln\left(K_{D}^{mut}/K_{D}^{WT}\right)$ with experimental errors in all cases reported as approximately 0.1 p*K*_D_ unit, that is, less than 0.1 kcal mol^−1^.^27^ [c] Binding affinity of the ligand to the (mutant) receptor was lower than the experimental threshold (p*K*_D_<5 in all cases). Errors are standard error of the mean (s.e.m.) over a total of 10 replicates.

Once the QresFEP protocol was validated to reproduce the BPM data on experimentally known structures, we moved on to the chromone scaffold, for which there is analogous BPM data, but no crystal structure available. Here, two reasonable binding modes were generated by molecular docking of **Chromone 14**. The two poses, denoted as A and B (see Figure 2), form at least one hydrogen bond with Asn253^6.55^ (a highly conserved interaction in AR ligand recognition)^23^ and are related by a symmetry axis along the bicyclic core of the chromone scaffold. According to the docking score function used, both poses were energetically equivalent (−9.30 vs. −9.17 for pose A and B respectively) making it difficult at this point to discern the correct one, in line with the previous binding hypothesis by Andrews et al.^30^, ^32^

In pose A (Figure 2 A), Asn253^6.55^ forms an H‐bond with the nitrogen in the 4‐methylthiazole group, leaving the carbonyl group potentially exposed to the internal water network stabilized by residues in TM7, as observed in the A_2A_AR crystal structure with **ZM241385** 26 (see Supporting Information, Figure S1). Conversely, in pose B (Figure 2 B) this carbonyl forms an additional hydrogen bond with N253^6.55^. We hypothesized that in principle, the symmetry axis would allow exchanging substituents at R^6^ and R^7^ between the binding poses, something that we would explore later in this work (see below).

Our strategy to select the most reliable binding mode was to compute the effect on ligand binding of the 8 mutations from the BPM panel^27^ for each pose, and compare the results with the experimental values. The results (Figure 2 C, Table 1 and Supporting Information, Table S1) highlight pose A as the binding mode with the best correlation to experimental data. A MAE of 0.50 kcal mol^−1^ was observed for this pose, which is comparable to the results obtained for the co‐crystallized antagonist **ZM241385**. In addition, the correlation coefficient of R^2^=0.74 falls between those observed for **ZM241385** and triazine **4 g**. Conversely, the corresponding values calculated on pose B are much higher (MAE=1.53 kcal mol^−1^) and the correlation is completely lost (R^2^=0.03). Additionally, the computed loss of binding affinity upon the N253^6.55^A mutation is closer to experiment in pose A than in pose B, indicating that the additional H‐bond between Asn253^6.55^ and the carbonyl in pose B would not contribute to more favorable binding free energies. From the MD simulations, we further observed that the binding modes are not related by a symmetry axis as initially hypothesized, and the substituents at positions R^6^ and R^7^ are exploring different positions in the binding site, making them no longer readily interchangeable.

### Design, Synthesis and Pharmacological Evaluation of Chromone Derivatives

**Scheme 1:**
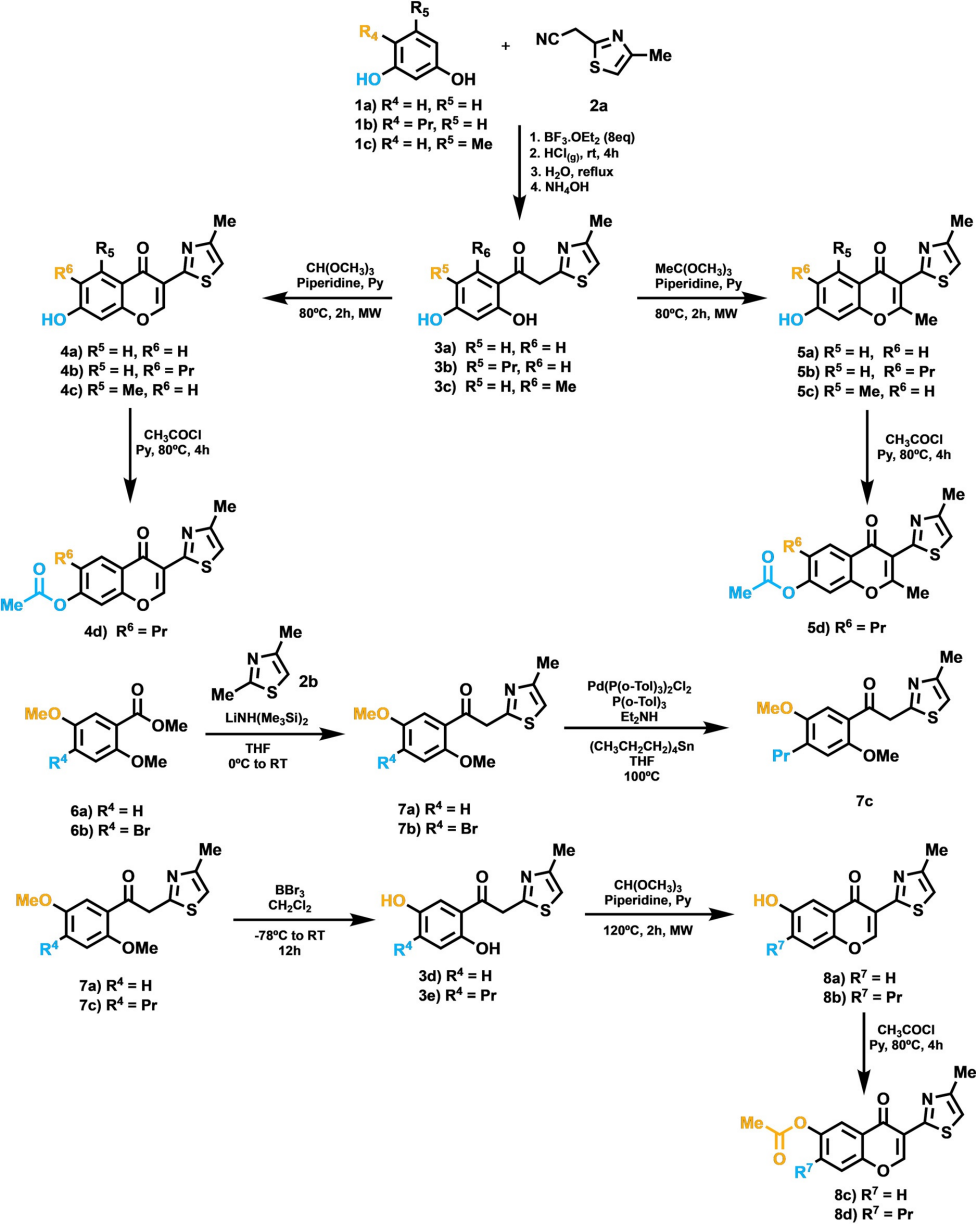
Structure and synthetic pathways employed to assembly chromones **4**, **5** and **8**.

On the basis of the binding mode hypothesis assessed by the FEP calculations, we designed a small collection of chromone derivatives (Scheme 1), aiming to systematically explore two different variables on A_2A_AR affinity: methylation at position 2 (series **5**) and the aforementioned effect of switching the oxygenated function (−OH or −OCOMe) from the original position 7 (series **4**) to position 6 (series **8**), swapping the alkyl substitution (Pr/H) accordingly. A collection of 11 molecules was prepared as depicted in synthetic Scheme 1. Briefly, the key 2‐hydroxyphenyl ketones **3** were obtained by either the Hoesch method^30^ or Claisen condensation,^36^ employing phenols **1** or esters **6** and thiazole derivatives **2 a** and **2 b** as reactive precursors, respectively. Treatment of ketones **3** with orthoesters (formate or acetate) enabled the efficient chromone core formation,^37^ thus defining the substituent pattern at position 2 (H or Me). Finally, the required acetates were prepared by reaction of the corresponding phenols with acetyl chloride. The full synthetic methodology is provided in the Supporting Information.

The affinity of these compounds for the WT A_2A_AR was then evaluated with the same SPR assay used to generate the BPM data, using the A_2A_AR‐STAR2 construct (see Supporting Information, Methods).^27^ The data confirmed that methylation at position 2 (compounds **5 a**–**d**) is generally unfavorable for binding, compared to the parent series (compounds **4 a**–**d**). This is in agreement with previous work by Andrews et al.,^30^ where methylation of the low affinity compound **4 a** (corresponding to chromone 8 as reported by Andrews et al.,^30^ with p*K*_i_=5.7) resulted in a relative decrease in affinity of 0.6 log units (Chromone 12, p*K*_i_=5.1). We observe a similar difference for this pair of compounds (Table 2, Δp*K*_D_=0.85), while this effect is amplified in the case of the high affinity precursor (see **4 d** as compared to **5 d**, Δp*K*_D_=2.7). More surprising was the observation of a similar effect when the substituents between R^6^ and R^7^ are swapped (compounds **8 a**–**d**), which again was most pronounced for the high affinity compound **4 d** (see Table 2), with a drop of affinity of 2.7 log units for the corresponding methylated derivative **8 d**.

**Table 2:** SPR affinity data for the series of Chromone derivatives synthesized in this work. 
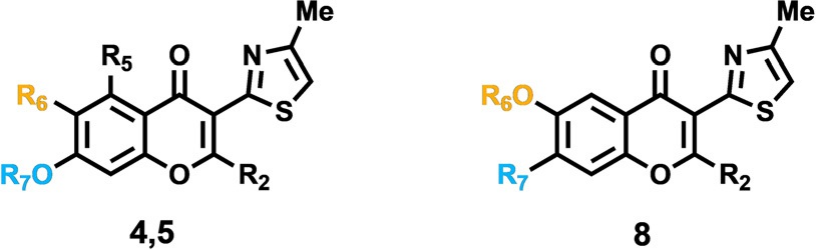

Compound	Substituents				p*K*_D_^[a]^
	R^2^	R^5^	R^6^	R^7^	
**4 a** ^[b]^	H	H	H	H	5.95
**4 b** ^[b]^	H	H	C_3_H_7_	H	6.20
**4 c**	H	CH_3_	H	H	5.80
**4 d** ^[b]^ (**Chromone 14**)	H	H	C_3_H_7_	COCH_3_	8.60
**5 a** ^[b]^	CH_3_	H	H	H	5.10
**5 b**	CH_3_	H	C_3_H_7_	H	5.42
**5 c**	CH_3_	CH_3_	H	H	5.36
**5 d**	CH_3_	H	C_3_H_7_	COCH_3_	5.90
**8 a**	H	H	H	H	5.50
**8 b**	H	H	H	C_3_H_7_	5.70
**8 d**	H	H	COCH_3_	C_3_H_7_	5.90

[a] Experimental errors in all cases reported as approximately 0.1 p*K*_D_ unit, that is, less than 0.1 kcal mol^−1^. [b] Compounds previously reported in ref. 30.

### Computational Evaluation of the Proposed Binding Modes

From the pharmacological data on the expanded series of chromones and the initial MD simulations, it appears that the two binding poses differ more than just purely on the rotation axis of the chromone scaffold.

**Figure 3:**
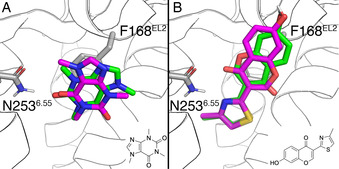
A) Dual binding mode of caffeine, as extracted from the A_2A_AR crystal structure with the A_2A_AR (PDB code 5MZP). Colour code is green (binding mode A) and magenta (binding mode B). B) modelled binding modes of **Chromone 4 a**, following the same colouring Scheme as in panel A.

The following step was then to investigate whether all compounds in the series would adopt the preferred binding mode A, or if binding mode B is accessible by some of the compounds, depending on the pattern of substitutions. Such a hypothesis was experimentally observed for caffeine, which in contrast to other xanthine derivatives presents a dual binding mode to the A_2A_AR (see Figure 3 A). Indeed, an isoenergetic dual binding mode for caffeine was previously hypothesized on the basis of free energy calculations,^38^ before experimental observation of dual‐occupancy crystal structures in complex with the A_2A_AR.^39^, ^40^ Conversely, the X‐ray structure of the N_7_‐demethylated analogue theophylline shows that this molecule adopts only one of these binding modes, where the acidic hydrogen at position N_7_ makes an additional H‐bond contact with N253^6.55^ (Figure 3). We thus evaluated whether our recently developed dual‐topology protocol QligFEP was suitable to capture this different behavior, by a direct estimation of relative binding free energies between the two poses, in analogy to the efficiency of this protocol to compare topologically unrelated ligands (e.g., scaffold hopping).^6^ The results for the A_2A_‐xanthine system shown in Table 3 show that this is the case: the negligible calculated free energy difference between the two poses for caffeine is in line with the equally populated dual binding mode in the crystal structure with the A_2A_AR (PDB code 5MZP, Figure 3 A). In contrast, the single binding mode observed in the crystal structure of theophylline (blue color in Figure 3 A) is energetically favored by 1.6 kcal mol^−1^. Subsequent application of the same strategy in the generated chromone series showed a similar energy gap between the two binding poses considered for the simplest chromone in our series (**4 a**, Figure 3 B), with pose A being 1.7 kcal mol^−1^ more favorable than pose B. Notably, this energy gap increases significantly for the highest affinity compound **4 d**, suggestive of an optimal anchoring of the R^6^ acetate and R^7^ n‐propyl substituents in pose A (Table 3).

**Table 3:** Calculated free energy difference between two alternative poses for A_2A_AR antagonists.

	Pose comparison A → B	Ligand perturbation Ligand 1 (H) → Ligand 2 (CH_3_)		
Ligand 1	ΔΔ*G*_calc_ [kcal mol^−1^]	Ligand 2	ΔΔ*G*_exp_ [kcal mol^−1^]	ΔΔ*G*_calc_ [kcal mol^−1^]^[a]^
Caffeine	0.47±0.49	–	–	–
Theophylline	1.59±0.87	Caffeine	0.6^[b]^	0.31±0.32
**4 a**	1.65±0.93	**5 a**	1.16±0.1^[c]^	2.06±0.72
**4 d**	8.76±0.81	**5 d**	3.68±0.1^[d]^	1.66±1.09

[a] Calculations performed in the selected pose A (see text). [b] Δ*G*_bind_ (caffeine—theophylline), extracted from ChEMBL.^41^ [c] Δ*G*_bind_ (**5 a**–**4 a**) and [d] Δ*G*_bind_ (**5 d**–**4 d**), data from Table 2.

We consequently retained the selected binding pose for each scaffold (i.e., xanthine and chromone) and computed the affinity shifts observed upon methylation. The calculations reproduced the experimental decrease in affinity observed for caffeine, as compared to the N_7_ demethylated analogue theophylline (Table 3). Similarly, the corresponding FEP calculations capture the experimentally observed negative effect on A_2A_ affinity due to methylation on R^2^ in the original chromones series **4** (see series **5**, see Table 2).^30^ The two pair comparisons performed (**4 a** → **5 a** and **4 d** → **5 d**, see Table 3) qualitatively capture this effect, which is experimentally more pronounced in the last case. Notably, a comparison of the endpoint configurations of the **4 d** → **5 d** FEP transformation provide the structural reason of this effect: to avoid the induced steric hindrance of the new methyl group with Asn253^6.55^, the core scaffold of **5 d** moved approximately 1.5 Å away from the original pose A of **4 d** while maintaining the interaction between the nitrogen in the 4‐methylthiazole group and the key asparagine residue.

### X‐Ray Crystallography

**Figure 4:**
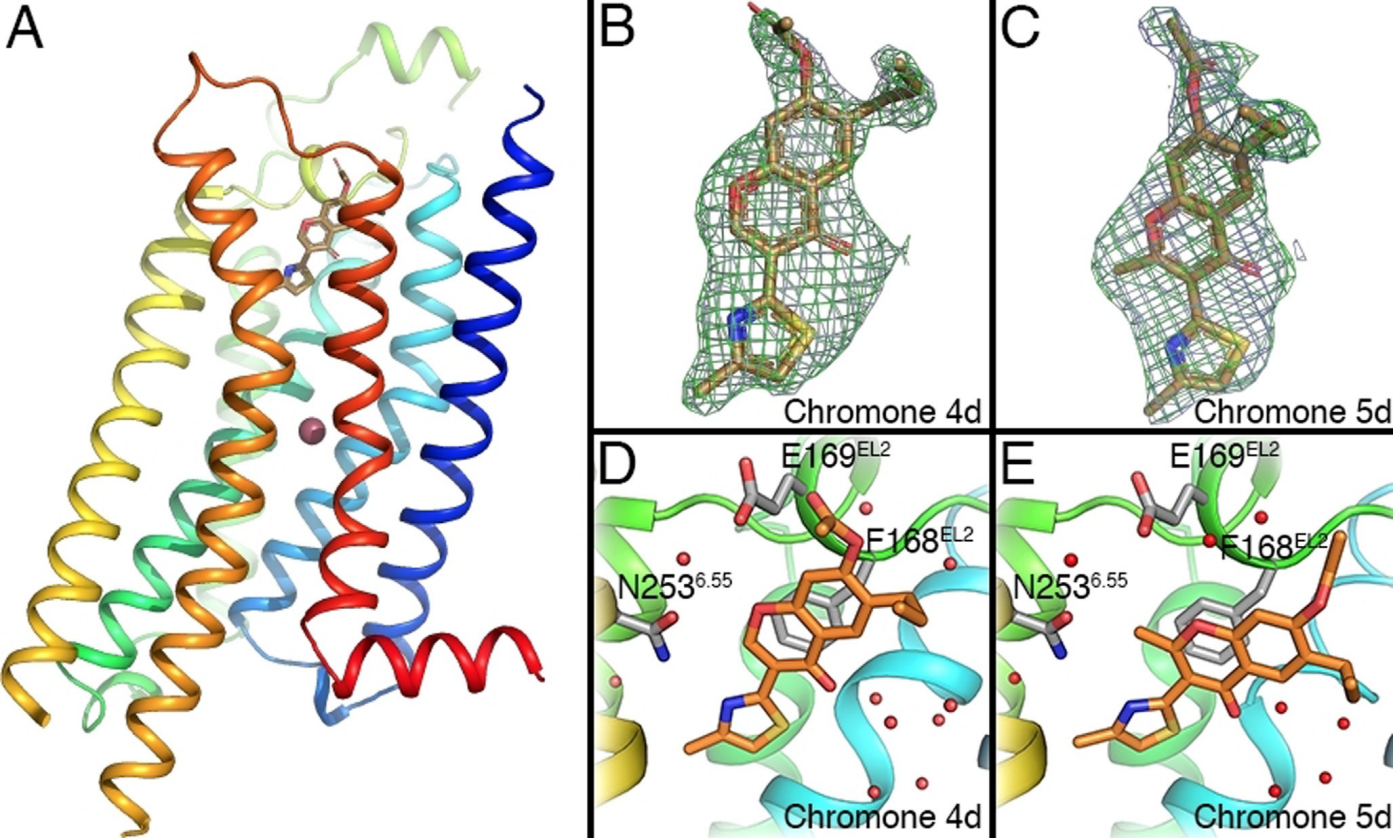
A) Crystal structures of the A_2A_AR and compound **4 d** (PDB code: 6ZDR), ligand shown in sticks and sodium ion shown as a sphere. Electron densities of chromones **4 d** (B) and **5 d** (C; PDB code: 6ZDV). Omit maps are 2 *F*_o_−*F*_c_ at 1 sigma (light blue mesh) and Fo‐Fc at 3 sigma (green mesh). Binding mode of compound **4 d** (D) and **5 d** (E); ligands and the conserved residue N253^6.55^ shown as sticks, water molecules in red spheres.

**Figure 5:**
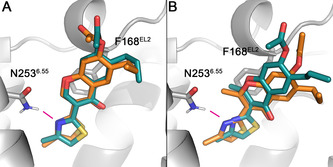
Crystal structure (orange) and modelled coordinates (cyan) of (A) the highest affinity compound **4 d** (PDB code: 6ZDR) and (B) the methylated derivative **5 d** (PDB code: 6ZDV) with the A_2A_AR. H‐bond interactions are indicated in magenta.

In an effort to unequivocally reveal the binding mode of the chromone scaffold, the A_2A_AR‐STAR2 construct^27^ was crystallized with compound **4 d** (PDB code: 6ZDR) and its 2‐methyl analogue **5 d** (PDB code: 6ZDV). The structures were obtained following the in meso soaking approach (see Supporting Information, Methods), and could be refined down to a resolution of 1.92 and 2.13 Å respectively. Statistics for data collection and refinement are given in the Supporting Information, Table S2. The overall structure of the A_2A_AR receptor is highly similar to previously solved structures with other antagonists (see Figure 4 A), with an RMSD of 0.46 Å for the Cα trace as compared to the **ZM241385** structure (PDB 4EIY, calculated using PyMOL^42^). The two structures show clear positive omit density at 1σ for the presence of the chromone compounds in the orthosteric binding site (Figure 4 B and C), in both cases adopting binding mode A (Figure 4 D and E). All residues in the binding site show a conserved rotameric state between the two structures and the A_2A_AR‐**ZM241385** complex. The only exception is Y271^7.36^, which slightly rotates outward served to accommodate the alkyl tail of the chromone at R^6^ (see Supporting Information, Figure S2A), an induced‐fit effect that was previously observed for other A_2A_AR ligands.^33^ The structure with the high affinity ligand **4 d** shows outstanding agreement with the computational model (see Figure 5 A) with an RMSD of 0.67 Å between the docked and experimental poses, and is indeed highly similar to previously reported binding modes of the compound.^30^, ^32^ The core of the scaffold is anchored by an H‐bond between the nitrogen in the 4‐methylthiazole ring (coplanar with the chromone scaffold) and N253^6.55^, and complemented by a π‐π stacking with F168^EL2^, in line with the binding pattern observed for many other A_2A_AR antagonists.^20^ The ester at position R^7^ is stabilizing the Glu169^5.30^–His264^7.29^ ionic pair, which closes the EL2‐EL3 interface, while the propyl at R^6^ makes contacts with Tyr271^7.53^ and Met270^7.52^. Given the good resolution of the structure, we could observe a well‐defined water network of the interface between the carbonyl moiety of the chromone core and the receptor (Figure 4 D). While most water positions are conserved as compared to the A_2A_AR‐**ZM241385** complex, ligand **4 d** displaces a number of water molecules observed in the first hydration shell around that ligand (see Supporting Information, Figure 2 B). Some of these waters were previously associated to a high‐energy or “unhappy” state,^30^ which could partially explain the high affinity of this particular compound.

The A_2A_AR‐**5 b** complex (Figure 4 E) further shows that methylation at position R^2^ displaces the chromone core by 1.79 Å, in accordance with observations from the FEP simulations (Table 3 and Figure 5 B, RMSD=1.24 Å between docked and experimental ligand configuration). The 4‐methylthiazole ring of compound **5 d** remains almost co‐planar with the chromone scaffold (torsional angle of 16 degrees), and the H‐bond interaction between the nitrogen in this ring and Asn253^6.55^ is maintained. Indeed, the methylation does not disrupt the position of Asn253^6.55^, which remains linked to Glu169^5.30^ via a conserved water molecule. Similarly, the water molecule deeper in the binding crevice remains, mediating a common anchoring point between the carbonyl group of the two ligands and His276^7.43^. However, the relative displacement of the chromone core on **5 d** situates the substituents at R^6^ and R^7^ in sub‐optimal pockets (Figure 4 D), displacing two of the water molecules described for compound **4 d**, while a new water molecule in the **5 d** complex replaces the role of the acetate in **4 d** (see Figure 5 D) in the stabilization of the Glu169^5.30^–His264^7.29^ ionic pair. This could explain why the inclusion of an acetyl moiety in compound **5 d** could not recover the affinity of the parent **5 b** (Δp*K*_D_=0.48, Table 2) as much as it was observed for the **4 b**/**4 d** pair (Δp*K*_D_=2.6). Finally, the crystal structures offered the opportunity to revise the **4 d** → **5 d** FEP transformation, which was somewhat underestimated (see Table 3). However, the calculated value using the starting pose from the experimental coordinates did not change significantly, (ΔΔ*G*_calc_=1.54±1.22 kcal mol^−1^) indicating that the docking pose was an accurate enough starting point for the FEP calculations.

## Conclusion

We describe a robust workflow to iteratively improve receptor‐ligand binding models, based on mapping of available experimental data onto structural information via free energy calculations. We applied this protocol to provide new insights in the binding mode of a recent series of A_2A_AR antagonists. An initial binding mode hypothesis was generated based on the exploration of BPM and SAR data of the original chromone series using FEP. This constituted the basis for the design, synthesis and evaluation of an expanded series of chromone derivatives. The experimental results were conveniently interpreted with the aid of a second iteration of FEP calculations, which reinforced the binding hypothesis. Finally, X‐ray crystallography experimentally confirmed this binding mode, supporting the rational design of these compounds. These structures, combined with the FEP calculations, provide structural and energetic insights in the determinants of high affinity binding of the chromone scaffold series. We expect the presented workflow to be of general applicability in structure‐based drug design, particularly in the case of GPCRs where structures of receptor‐ligand complexes are increasingly available.

## Conflict of interest

The authors declare no conflict of interest.

## Supporting information

As a service to our authors and readers, this journal provides supporting information supplied by the authors. Such materials are peer reviewed and may be re‐organized for online delivery, but are not copy‐edited or typeset. Technical support issues arising from supporting information (other than missing files) should be addressed to the authors.

Supplementary
