# Supplementary material for: X‐Ray Crystallography and Free Energy Calculations Reveal the Binding Mechanism of A2A Adenosine Receptor Antagonists
Source: Angew Chem Int Ed Engl. 2020 Jul 22;59(38):16536–43. doi: 10.1002/anie.202003788 (PMC7540567; doi:10.1002/anie.202003788)
Supplement: Supplementary file 1 — Supplementary [file ANIE-59-16536-s001.pdf]

Angewandte Chemie
Eine Zeitschrift der Gesellschaft Deutscher Chemiker

Supporting Information

X-Ray Crystallography and Free Energy Calculations Reveal the Binding Mechanism of A_{2A} Adenosine Receptor Antagonists**

*Willem Jespers, Grégory Verdon, Jhonny Azuaje, Maria Majellaro, Henrik Keränen, Xerardo García-Mera, Miles Congreve, Francesca Deflorian, Chris de Graaf, Andrei Zhukov, Andrew S. Doré, Jonathan S. Mason, Johan Åqvist, Robert M. Cooke, Eddy Sotelo, and Hugo Gutiérrez-de-Terán**

anie_202003788_sm_miscellaneous_information.pdf

Author Contributions

W.J. Conceptualization: Lead; Data curation: Lead; Formal analysis: Lead; Investigation: Lead; Methodology: Equal; Writing—Original Draft: Lead; Writing—Review & Editing: Supporting
G.V. Formal analysis: Supporting; Investigation: Equal; Writing—Review & Editing: Supporting
J.A. Formal analysis: Equal; Investigation: Equal
M.M. Formal analysis: Equal; Investigation: Supporting; Methodology: Supporting
H.K. Conceptualization: Supporting; Investigation: Equal; Methodology: Supporting
X.G. Formal analysis: Supporting; Supervision: Supporting
M.C. Supervision: Supporting; Writing—Review & Editing: Supporting
F.d. Formal analysis: Supporting
C.d. Conceptualization: Supporting; Formal analysis: Supporting; Writing—Review & Editing: Supporting
A.Z. Supervision: Supporting
A.D. Supervision: Supporting
J.M. Conceptualization: Supporting; Project administration: Supporting; Writing—Review & Editing: Supporting
J.Å. Conceptualization: Supporting; Funding acquisition: Lead; Methodology: Supporting; Supervision: Supporting; Writing—Review & Editing: Supporting
R.C. Conceptualization: Supporting; Formal analysis: Supporting; Funding acquisition: Supporting; Supervision: Supporting; Writing—Review & Editing: Supporting
E.S. Conceptualization: Supporting; Methodology: Equal; Project administration: Equal; Writing—Original Draft: Supporting; Writing—Review & Editing: Equal
H.G. Conceptualization: Lead; Formal analysis: Equal; Supervision: Lead; Validation: Supporting; Writing—Original Draft: Equal; Writing—Review & Editing: Lead.

Abstract: We present a robust protocol based on iterations of free energy perturbation (FEP) calculations, chemical synthesis, biophysical mapping and X-ray crystallography to reveal the binding mode of an antagonist series to the adenosine A_{2A} receptor (AR). Eight A_{2A}AR binding site mutations from biophysical mapping experiments were initially analysed with sidechain FEP simulations, performed on alternate binding modes. The results distinctively supported that one binding, which was subsequently used to design new chromone derivatives. Their affinities for the A_{2A}AR were experimentally determined and investigated through a cycle of ligand-FEP calculations, validating the binding orientation of the different chemical substituents proposed. Subsequent X-ray crystallography of the A_{2A}AR with a low and a high affinity chromone derivative confirmed the predicted binding orientation. The new molecules and structures here reported were driven by free energy calculations, and provide new insights on antagonist binding to the A_{2A}AR, an emerging target in immuno-oncology

DOI: 10.1002/anie.202003788

Table of Contents

Experimental Procedures 3
- Computational Methods 3
- Chemistry. 5
- Protein purification and SPR. 8
- Crystallization. 9

Figure S1. 11
Figure S2. 12
Table S1 13
Table S2 14
Spectroscopic Data of Compounds 4a-d, 5a-d and 8a-d: 15
Spectroscopic Data for the Key Intermediates 3a-e and 7a-c 17
References 19

Experimental Procedures

Computational Methods

We used our generalized FEP protocol for amino acid mutations, QresFEP,[1–3] to estimate the effects of single point mutations on ligand binding. The high-resolution crystal structure of the A_2AR with antagonist **ZM241385** (4EIY[4]) was used as receptor starting structure, after modeling the missing loops (C-terminal fragment of EL2 and most of EL3), proton addition and removal of the engineered BRIL fusion protein, as described elsewhere.[5] The 3D coordinates for the ligands were generated with LigPrep and subsequently docked to the prepared $A_{2A}AR$ structure using Glide.[6] In case of **Triazine 4g**, the obtained results were compared to the available crystal structure (3UZA[7]) and the best pose maintained for further analysis. An apo reference structure, based on the 4EIY crystal structure, was generated by removing the ligand, but keeping crystallographic waters, and subsequently embedded in a solvated membrane environment using PyMemDyn[8]. Shortly, this protocol embeds a structure in a pre-equilibrated POPC (1- palmitoyl-2-oleoyl phosphatidylcholine) membrane model such that the TM bundle is parallel to the vertical axis of the membrane. The system is then soaked with bulk water and inserted into a hexagonal prism-shaped box that is energy-minimized and carefully equilibrated during 5 ns, following the PyMemDyn protocol described elsewhere.[8–10] The standard OPLS all-atom (OPLS-AA) force field is used for all residues,[11,12] and parameters for membrane lipids were taken from the Berger united-atom model.[13] The corresponding equilibrated holo structures were generated by restoring the docked ligands and removing overlapping water molecules.

The receptor-ligand structures in the equilibrated membrane were then transferred to the MD package Q, in order to perform FEP calculations under spherical boundary conditions.[14,15] A 50 Å diameter sphere was centered on the center of geometry of **ZM241385** (or equivalent point in the remaining structures), where solvent atoms are subject to polarization and radial restrains using the surface constrained all-atom solvent (SCAAS) model to mimic the properties of bulk water at the sphere surface. [15,16] Atoms lying outside the simulation sphere are tightly constrained (200 kcal/mol/$Å^2$ force constant) and excluded from the calculation of non-bonded interactions. Within the simulation sphere, long range electrostatics

interactions beyond a 10 Å cut off were treated with the local reaction field method, [17] except for the atoms undergoing the FEP transformation where no cutoff was applied. Solvent bond and angles were constrained using the SHAKE algorithm.[18] All ionizable residues outside the sphere and those within the boundary were considered in their neutral form as described elsewhere.[19] Residue parameters were translated from the latest version of the OPLSAA/M force field,[20] whereas ligand OPLS2005 parameters were retrieved from Schrodinger's ffld_server,[21] and translated to Q following the QligFEP protocol.[19] The simulation sphere was warmed up from 0.1 to 298 K during a first equilibration period of 0.61 nanoseconds, where the initial restraint of 25 kcal/mol/Å^2 imposed on all heavy atoms was slowly released. Thereafter the system was subject to unrestrained MD simulations, starting with a 0.25 nanosecond unbiased equilibration period which is followed by the FEP sampling, where atom transformations occur between initial and ending states, evenly divided into a number of λ windows (see below). This sampling is replicated on 10 independent MD simulations with different initial velocities, each of them consisting in 10 ps sampling per λ window using a 1 fs time step in all cases.

These MD parameters were adopted for both sidechain and ligand perturbations, in separate protocols: The single-topology FEP protocol for amino acid perturbations, QresFEP,[3] divides the sidechain perturbation to alanine into separate stages, where atom annihilations occur gradually for each charge group (as defined on the OPLS force field), starting from the most topologically distant from the C_β atom:[1,2] the partial charges are initially removed, after which the van der Waals potentials are transformed into smoother soft-core potentials, before the annihilation of the corresponding group of atoms, and finally restoring the partial charges of the final species. The number of perturbation stages needed for the full annihilation depends on the nature of the sidechain involved, ranging from four (Ser) to nine (Trp), where each of the subsequent stages is evenly divided into 20 λ windows. To fulfill a thermodynamic cycle, the same sidechain annihilation is simulated in the apo structure of the protein, so that the energetic difference between these two processes equals the binding affinity shift due to the mutation.

All ligand perturbations were performed with our dual-topology QligfFEP protocol,[19] where the full transformation of one ligand into another is performed along a linear λ sampling consisting of 100 windows. The reference state to create the thermodynamic cycle is in this case the MD simulation of the ligands' transformation in an equivalent sphere of water, and the in this case difference between the protein-bound and reference ligand transformations equals the difference in binding affinity between the two ligands.

In both residue and ligand transformations, relative binding free energies were estimated by solving the thermodynamic cycle utilizing the Bennet acceptance ratio method (BAR)[22] as

$$\Delta G_i = -\beta^{-1} ln \frac{\langle 1+e^{-\beta(\Delta U \Delta \lambda_i - C_i)}\rangle_{i+1}}{\langle 1+e^{+\beta(\Delta U \Delta \lambda_i - C_i)}\rangle_i} + C_i$$

where the constants C_i are optimized iteratively so that the two ensemble averages become equal, yielding $\Delta G_i = C_i$.

Chemistry.

Unless otherwise indicated, all starting materials, reagents and solvents were purchased and used without further purification. After extraction from aqueous phases, the organic solvents were dried over anhydrous sodium sulphate. The reactions were monitored by thin-layer chromatography (TLC) on 2.5 mm Merck silica gel GF 254 strips, and the purified compounds each showed a single spot; unless stated otherwise, UV light and/or iodine vapour were used to detect compounds. Most reactions were performed in coated Kimble vials on a PLS (6×4) Organic Synthesizer with orbital stirring. The purity and identity of all tested compounds were established by a combination of mass spectrometry, NMR spectroscopy and HPLC as described below. Purification of isolated products was carried out by column chromatography (Kieselgel 0.040–0.063 mm, E. Merck) or medium pressure liquid chromatography (MPLC) on a CombiFlash Companion (Teledyne ISCO) with RediSep pre-packed normal-phase silica gel (35–60 µm) columns followed by recrystallization. Melting points were determined on a Gallenkamp melting point apparatus and are uncorrected. The NMR spectra were recorded on Bruker AM300 and XM500

spectrometers. Chemical shifts are given as δ values against tetramethylsilane as internal standard and *J* values are given in Hz. Mass spectra were obtained on a Varian MAT-711 instrument. High-resolution mass spectra were obtained on an Autospec Micromass spectrometer. Analytical HPLC was performed on an Agilent 1100 system using an Agilent Zorbax SB-Phenyl, 2.1 mm × 150 mm, 5 µm column with gradient elution using the mobile phases (A) H_2O containing 0.1% CF_3COOH and (B) MeCN and a flow rate of 1 mL/min. The purity of all tested compounds was determined to be >95%.

General procedure for the synthesis of 1-(2,4-dihydroxyphenyl)-2-(4-methylthiazol-2-yl)ethan-1-ones 3a-c: The corresponding resorcinol derivative **1a-c** (4 mmol) and 2-(4-methylthiazol-2-yl)acetonitrile **2a** (4 mmol) were dissolved in 9 mL of 46.5% $BF_3.O(C_2H_5)_2$ and the mixture was stirred for 10 minutes at room temperature. Dry HCl gas was bubbled through the mixture for 2h at 25ºC and the resulting mixture was stirred for 4h at room temperature, diluted with water (100 mL) and the refluxed for 1h. The pH of the solution was adjusted to 3 with 30% ammonia solution and the resulting yellow precipitate was filtered off and washed 3 times with water. The crude precipitate **3a-c** was used without any further purification in the next step.

General procedure for the synthesis of 7-hydroxy-3-(4-methylthiazol-2-yl)-4*H*-chromen-4-ones 4a-c: The corresponding ketone **3a-c** (0.8 mmol) was dissolved in 2 mL of pyridine, treated with trimethyl orthoformate (8 mmol) and piperidine (0.3 mmol). The reaction mixture was heated at 80 ºC under microwave irradiation for 2 h. The solvent was removed under reduced pressure to give an oil residue, which was purified by flash chromatography on silica gel using hexane/AcOEt mixtures affording the products in satisfactory yield (45 – 62%).

General procedure for the synthesis of 7-hydroxy-2-methyl-3-(4-methylthiazol-2-yl)-4H-chromen-4-ones 5a-c: In a round bottom flask the intermediate **3a-c** (0.8 mmol) was dissolved in 2 mL of pyridine, treated with triethyl orthoacetate (8 mmol) and piperidine (0.3 mmol). The reaction mixture was heated at 80ºC under microwave irradiation for 2h. The solvent was removed under reduced pressure to give an oil residue, which was purified by flash chromatography on silica gel using hexane/AcOEt mixtures affording the products in satisfactory yield (40 – 59%).

General procedure for the synthesis of the acetyl derivatives 4d and 5d: To a solution of **4a** (0.15 mmol) or **5a** (0.15 mmol) in 2 mL of pyridine was added acetyl chloride (1.5 mmol). The reaction mixture was heated at 80ºC for 4h. The solvent was removed under reduced pressure to give an oil residue, which was purified by flash chromatography on silica gel using hexane/AcOEt mixtures affording the products in good yield (79 – 83%).

General procedure for the synthesis of 1-(2,5-dimethoxyphenyl)-2-(4-methylthiazol-2-yl)ethan-1-ones 7a-b: To a solution of **6a-b** (5 mmol) and **2b** (5 mmol) in 50 mL of dry THF (purged with argon) and cooled at -10ºC, was added dropwise a solution 1M of $LiN(Me_3Si)_2$ (7.5 mmol) in THF. The reaction mixture was stirred at room temperature for 12 h, then quenched with a mixture of saturated aqueous NH_4Cl solution (25 mL) and 25 mL of H_2O, extracted with CH_2Cl_2 (3x25 mL) and dried with Na_2SO_4. After filtration the solvent was removed under reduce pressure to afford a residue which was purified by flash chromatography on silica gel using hexane/AcOEt mixtures affording the products in satisfactory yield (43 – 50%).

General procedure for the synthesis of 1-(2,5-dimethoxy-4-propylphenyl)-2-(4-methylthiazol-2-yl)ethan-1-one 7c: A mixture of **7b** (2.5 mmol), $Pd(P(o\text{-}Tol)_3)_2Cl_2$ (0.025 mmol), $P(o\text{-}Tol)_3$ (0.05 mmol) and Et_2NH (7.5 mmol) under atmosphere of argon, were dissolved in 15 mL of dry THF in a sealed tube and stirred for 15 min, followed by the addition of $(CH_3CH_2CH_2)_4Sn$ (7.5 mmol), the reaction mixture was heated at 100ºC for 12h. The solvent was removed under reduce pressure and the residue was purified by flash chromatography on silica gel using hexane/AcOEt mixtures affording the products in high yield (80%).

General procedure for the synthesis of 1-(2,5-dihydroxyphenyl)-2-(4-methylthiazol-2-yl)ethan-1-ones 3d-e: In a round bottom flask the intermediate **7a-c** (2.0 mmol) was dissolved in 40 mL of dry CH_2Cl_2 under atmosphere of argon and cooled at -78ºC. A solution 1M of BBr_3 (6 mmol) in dry CH_2Cl_2 was added dropwise, the reaction mixture was stirred from -78ºC to room temperature for 12h and followed by the addition of 50 mL of water, the resulting yellow precipitate was filtered off and washed 3 times with water. The crude precipitate **3d-e** was used without any further purification in the next step.

General procedure for the synthesis of 6-hydroxy-3-(4-methylthiazol-2-yl)-4*H*-chromen-4-ones 8a-b: In a round bottom flask the corresponding intermediate **3d-e** (0.8 mmol) was dissolved in 2 mL of pyridine, treated with triethyl orthoformate (8 mmol) and piperidine (0.3 mmol). The reaction mixture was heated at 120ºC under microwave irradiation for 2h. The solvent was removed under reduced pressure to give an oil residue, which was purified by flash chromatography on silica gel using hexane/AcOEt mixtures affording the products in satisfactory yield (49 – 51%).

General procedure for the synthesis of the acetyl derivative 8c-d: To a solution of **8a-b** (0.15 mmol) in 2mL of pyridine was added acetyl chloride (1.5 mmol). The reaction mixture was heated at 80ºC for 4h. The solvent was removed under reduced pressure to give an oil residue, which was purified by flash chromatography on silica gel using hexane/AcOEt mixtures affording the products in good yield (74%).

Protein purification and SPR.

The structures of the human A_{2A} adenosine receptor in complexes with the chromones were obtained by following experimental protocols described previously.[31,32] Briefly, the A_{2A} receptor expression construct has been extensively engineered, and features truncations, thermostabilising mutations, and is fused to the Apocytochrome b_{RIL}562 in ICL3, and to a decahistidine tag at the C-terminus. The construct was expressed using the Bac to Bac Expression System (Invitrogen) in *Trichoplusa ni* Tni PRO cells, and the protein was purified at 4°C in decyl-β-maltopyranoside, and in the presence of 1 mM of Theophylline, using metal affinity (Ni-NTA Superflow, QIAGEN) and size exclusion chromatography (Superdex200, GE Healthcare).

SPR Assays were performed on a Biacore T200 instrument at 25ºC using sensor chip NiD 500m (Xantec Bioanalytics). The running buffer was 10 mM phosphate, pH 7.4, 2.7 mM KCl, 137 mM NaCl, 0.05 mM EDTA, 0.1% DDM, 5% DMSO.

The stabilised A_{2A} receptor was capture-coupled on the chip surface as follows: The surface was loaded with nickel by sequential 1-min injection of 350 mM EDTA and 0.5 mM $NiCl_2$. The surface was then activated for amine coupling using the amine coupling kit (GE Healthcare) and the receptors were

injected at 0.2 μM for 7 min. The surface was left overnight at standby flow before the analysis. Unmodified surface was used as a reference.

A blank sample and five concentrations of each compound in the range 0.5-8 μM were injected with a contact and dissociation time of 1 min. The reference and blank subtracted sensorgrams were fitted to 1:1 interaction model using the Biacore evaluation software (GE Healthcare) to obtain the association and dissociation rate constants and the affinity dissociation constant. If the rate constants were too high and beyond the Biacore evaluation range the steady state affinity alone was evaluated.

Crystallization.

Crystallization experiments were carried out in lipidic cubic phase at 20°C. Monoolein (Nu-Chek) supplemented with cholesterol (Sigma Aldrich) (ratio of 9:1 w/w) and 10 μM theophylline was mixed with the protein at a concentration of ~35mg.ml^{-1} (DC Protein Assay, Bio-Rad) using the twin-syringe method[23,24] and at a ratio of 4:6 (v/v). Boli were dispensed onto Laminex Glass Bases (Molecular Dimensions ltd) using a Mosquito LCP crystallization robot (TTP Labtech) and overlaid with 800 nL of crystallization solution consisting of 26-32% PEG 400, 0.1M Tri Sodium Citrate, pH = 4.7 to 5.4, 0.05M Sodium Thiocyanate, 2 % 2,5-Hexanediol, and 0.5 mM Theophylline, and sealed using Laminex Film covers (Molecular Dimensions Ltd). ~200μm long plate-shaped crystals grew within a week. Complexes with the chromones were obtained by soaking crystals *in situ*. Wells with crystals were filled through incision in cover with mother liquor without Theophylline but supplemented by 1 mM of a chromone, resealed with tape, incubated overnight at 20 °C, and flash-frozen in liquid nitrogen without the addition of further cryoprotectant.

X-ray diffraction data (0.25˚/ frame; ~500 frames per crystal) were collected on a Pilatus 6M detector at beamline I24 (Diamond Light Source) for crystals of the complex with **4d**, and on an Eiger 16M detector at beamline X06SA (Swiss Light Source) for crystals of the complex with **5d**. Swipes of data from individual crystals were integrated using *XDS*,[25] and merged and scaled together using *AIMLESS*[26]

from the CCP4 suite[27] and then further pipelined through the anisotropic scaling program STARANISO[28]. The final data reduction statistics are presented in Supporting Information Table S2.

The structures of the A_{2A}-StaR2-b_{RIL}562-chromone complexes were solved by molecular replacement (MR) with *Phaser*[29] using the previously reported A_{2A}-StaR2-b_{RIL}562-theophylline complex structure[30] as the search model (PDB code: 5MZJ). Model refinement was performed using *phenix.refine*,[31] and further using BUSTER (Global Phasing Limited, Cambridge, UK),[32] including TLS refinement for 2 groups corresponding to the receptor and b_{RIL}562 respectively. Refinement statistics are presented in Supporting Information Table S2. Structure figures were generated using PyMOL.[33]

Figure S1.

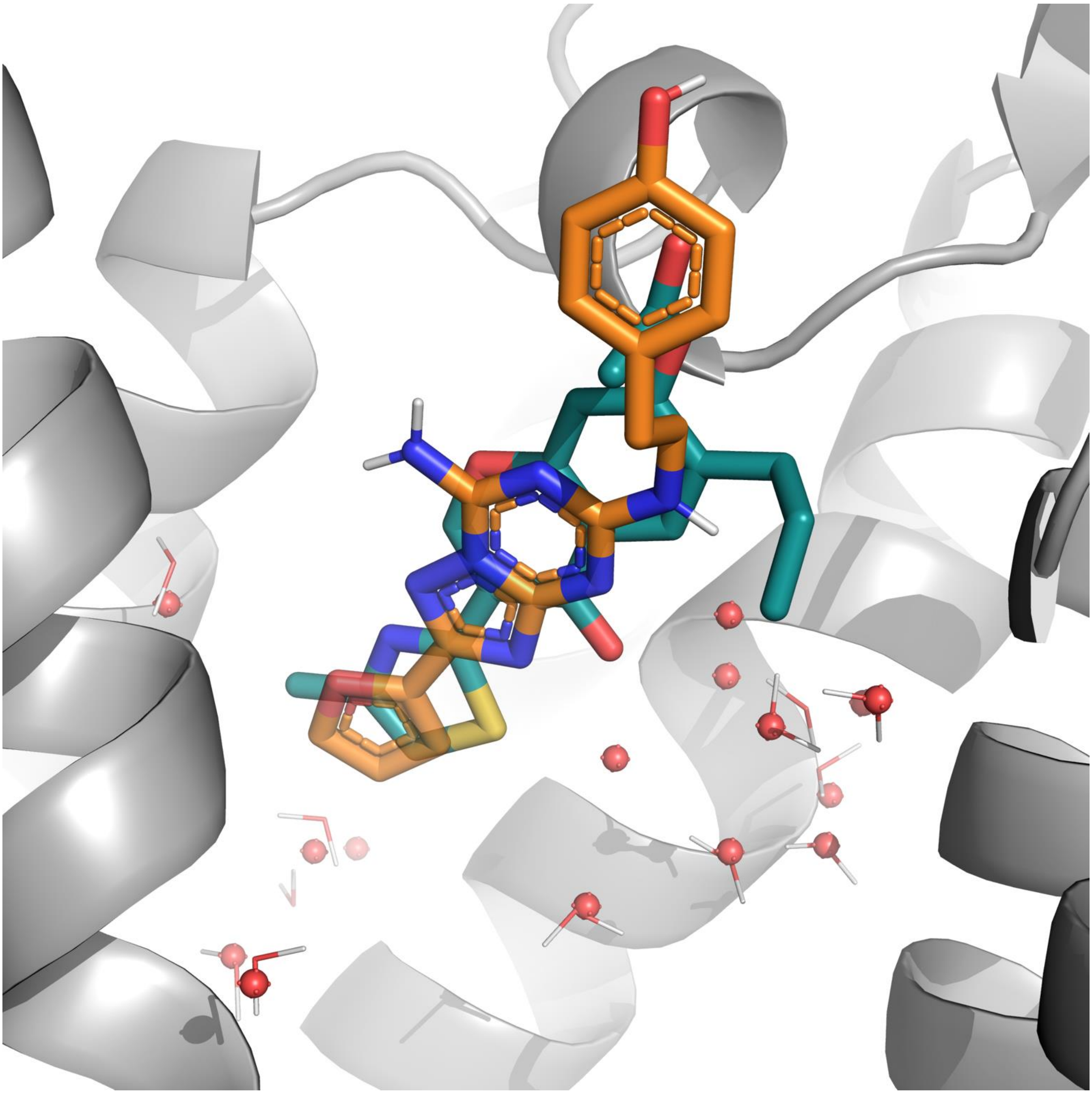

Overlay of the docking pose A of chromone **4d** (cyan sticks) as modelled in the binding site of the A_{2A}AR-**ZM241385** crystal structure (PDB code 4EIY, ligand depicted in orange sticks). The water molecules belonging to the 4EIY crystal structure are depicted in spheres, while the modelled water molecules from the A_{2A} AR-4d complex are indicated with sticks, showing the equivalence of many of these waters in the two complexes.

Figure S2.

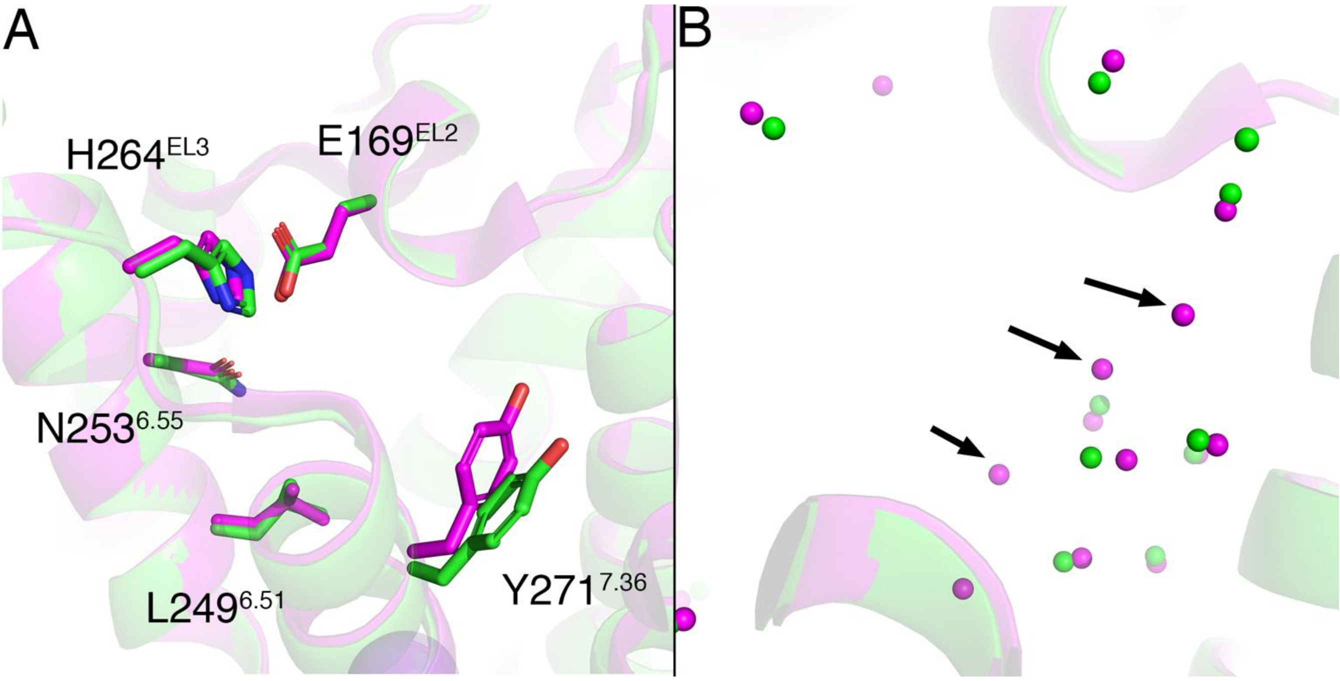

Two insets into the overlay of the binding site of the $A_{2A}AR$ as crystallized with the chromone **4d** (green structure, PDB code 6ZDR) or with **ZM241385** (magenta, PDB code 4EIY). Panel (A) highlights the residues in the binding site (in sticks); panel (B) shows the water molecules (spheres) belonging to either experimental complex with the same colour code with arrows indicating the waters in the first hydration shell displaced by chromone **4d**.

Table S1.

Experimental and calculated binding affinities for two chromone poses for the mutant receptor constructs reported in [34]. The free energies ($\Delta\Delta G$, kcal/mol) are extracted from the corresponding Ki values, using the relationship ($\Delta\Delta G_{bind}^{exp} = RTln(K_i^{mut}/K_i^{WT}$). The asterisk indicates that the binding affinity of the ligand to the (mutant) receptor was lower than the experimental threshold (pK_D < 5 in all cases).Errors are standard errors of the mean over a total of 10 replicates, experimental errors were reported as approximately 0.1 pK_D unit. [34]

	$\Delta\Delta G_{bind}$ (kcal/mol)		
Mutant	experiment	Pose A	Pose B
I66A	0.95	-0.08 ± 0.49	-1.29 ± 0.43
L85A	1.77	0.57 ± 0.54	-0.36 ± 0.49
L167A	0.14	0.44 ± 0.40	0.79 ± 0.41
M177A	-0.14	-0.30 ± 0.55	0.25 ± 0.60
N181A	0.41	0.72 ± 0.61	-1.91 ± 0.69
N253A*	3.14	3.49 ± 0.54	1.32 ± 0.47
Y271A	0.95	1.45 ± 0.76	2.56 ± 0.68
S277A	-0.55	-0.43 ± 0.44	0.49 ± 0.44
R^2		0.74	0.03
MAE		0.50	1.53

Table S2.

Crystallographic data collection and refinement statistics

Data collection	A_{2A}-chromone 4d	A_{2A}-chromone 5d
Number of crystals	2	2
Space group	$C222_1$	$C222_1$
Cell dimensions		
a, *b*, *c* (Å)	39.26, 180.23, 140.00	39.41, 179.83, 140.09
α, β, γ (°)	90, 90, 90	90, 90, 90
PDB code	6ZDR	6ZDV
Resolution (Å)	45.06-1.92 (2.04-1.92)	42.81-2.13 (2.24-2.13)
No. reflections	32704 (1521)	25465 (1268)
R_{pim}	0.052 (0.581)	0.064 (0.836)
$I/\sigma(I)$	9.2 (1.4)	11.0 (1.5)
$CC_{1/2}$	0.926 (0.527)	0.998 (0.458)
Completeness (%)		
spherical	84.9 (24.0)	89.2 (31.1)
ellipsoidal	90.8 (38.6)	91.8 (37.9)
Redundancy	6.9 (8.3)	9.4 (10.3)
**Refinement**		
Resolution (Å)	45.06-1.92	42.81-2.13
R_{work} / R_{free}	0.208/0.218	0.202/0.220
No. atoms	3389	3344
Protein	2958	2978
Ligand	24	25
Other	407	341
B factors		
Protein	47.47	50.19
Ligand	34.15	33.76
Other	49.52	50.28
R.m.s. deviations		
Bond lengths (Å)	0.022	0.022
Bond angles (°)	1.470	1.610

Ramachandran plot statistics:

- A2A-chromone 4d (residues in Preferred regions: 368/98.66%; Allowed regions: 5/1.34%; Outliers: 0/0.00%)
- A2A-chromone 5d (residues in Preferred regions: 369/97.88%; Allowed regions: 8/2.12%; Outliers: 0/0.00%)

Spectroscopic Data of Compounds 4a-d, 5a-d and 8a-d:

7-hydroxy-3-(4-methylthiazol-2-yl)-4*H*-chromen-4-one (4a): 128.5 mg, 62% yield, Mp 270 – 271ºC. ^{1}H NMR (300 MHz, DMSO-d_6) δ (ppm): 10.98 (s, 1H), 9.10 (s, 1H), 8.03 (dd, *J* = 8.7, 1.5 Hz, 1H), 7.32 (s, 1H), 7.00 (dd, *J* = 8.8, 2.3 Hz, 1H), 6.95 (d, *J* = 2.1 Hz, 1H), 2.41 (s, 3H). ^{13}C NMR (75 MHz, DMSO-d_6) δ (ppm): 178.2, 168.3, 162.4, 161.2, 159.9, 156.2, 132.4, 122.1, 121.1, 121.1, 120.8, 107.7, 22.0. HRMS (APCI) *m/z* cald. For $C_{13}H_{10}NO_3S$ $[M+1]^+$: 260.0376, found: 260.0341.

7-hydroxy-3-(4-methylthiazol-2-yl)-6-propyl-4*H*-chromen-4-one (4b): 108.4 mg, 45% yield, Mp 266 – 267ºC. ^{1}H NMR (300 MHz, DMSO-d_6) δ (ppm): 10.97 (s, 1H), 9.07 (s, 1H), 7.84 (s, 1H), 7.29 (s, 1H), 6.95 (s, 1H), 2.72 – 2.52 (m, 2H), 2.40 (s, 3H), 1.73 – 1.44 (m, 2H), 0.90 (t, *J* = 7.3 Hz, 3H). ^{13}C NMR (75 MHz, DMSO-d_6) δ (ppm): 178.1, 166.5, 161.4, 160.8, 159.8, 156.2, 134.5, 130.9, 122.0, 121.1, 120.5, 107.0, 36.5, 27.2, 22.0, 18.9. HRMS (APCI) *m/z* cald. For $C_{16}H_{16}NO_3S$ $[M+1]^+$: 302.0845, found: 3021.0844.

7-hydroxy-5-methyl-3-(4-methylthiazol-2-yl)-4*H*-chromen-4-one (4c) (1d): 111.4 mg, 51% yield, Mp 271 – 272ºC. ^{1}H NMR (300 MHz, DMSO-d_6) δ (ppm): 10.81 (s, 1H), 8.99 (s, 1H), 7.28 (s, 1H), 6.77 (d, *J* = 2.3 Hz, 1H), 6.73 (d, *J* = 2.5, 1H), 2.74 (s, 3H), 2.40 (s, 3H). ^{13}C NMR (75 MHz, DMSO-d_6) δ (ppm): 180.0, 166.9, 163.8, 161.4, 158.8, 156.1, 150.6, 147.6, 122.8, 121.0, 119.4, 106.1, 27.9, 22.0. HRMS (APCI) *m/z* cald. For $C_{14}H_{12}NO_3S$ $[M+1]^+$: 274.0532, found: 274.0521.

3-(4-methylthiazol-2-yl)-4-oxo-6-propyl-4*H*-chromen-7-yl acetate (4d): 40.6 mg, 79% yield, Mp 170 – 171ºC. ^{1}H NMR (300 MHz, $CDCl_3$-*d*) δ (ppm): 9.08 (s, 1H), 8.22 (s, 1H), 7.33 (s, 1H), 7.02 (s, 1H), 2.71 – 2.56 (m, 2H), 2.50 (s, 3H), 2.38 (s, 3H), 1.82 – 1.47 (m, 2H), 0.97 (t, *J* = 7.3 Hz, 3H). ^{13}C NMR (75 MHz, $CDCl_3$-*d*) δ (ppm): 174.2, 168.5, 156.5, 154.9, 154.5, 153.2, 151.7, 133.5, 127.2, 121.5, 118.3, 115.8, 111.9, 31.9, 22.7, 20.9, 17.0, 13.8. HRMS (APCI) *m/z* cald. For $C_{18}H_{18}NO_4S$ [M+1]+: 344.0951, found: 344.0952.

7-hydroxy-2-methyl-3-(4-methylthiazol-2-yl)-4*H*-chromen-4-one (5a): 117.9 mg, 54% yield, Mp 277 – 278ºC. ^{1}H NMR (300 MHz, DMSO-d_6) δ (ppm): 10.88 (s, 1H), 7.97 (d, J = 8.7 Hz, 1H), 7.31 (s, 1H), 7.01 – 6.90 (m, 1H), 6.87 (s, 1H), 2.89 (s, 3H), 2.42 (s, 3H). ^{13}C NMR (75 MHz, DMSO-d_6) δ (ppm): 178.9, 172.1, 168.2, 162.7, 161.7, 155.5, 132.4, 120.9, 120.7, 119.8, 119.7, 107.1, 26.4, 22.2. HRMS (APCI) *m/z* cald. For $C_{14}H_{12}NO_3S$ [M+1]$^+$: 274.0532, found: 274.0513.

7-hydroxy-2-methyl-3-(4-methylthiazol-2-yl)-6-propyl-4*H*-chromen-4-one (5b): 100.8 mg, 40% yield, Mp 228 – 229ºC. ^{1}H NMR (300 MHz, DMSO-d_6) δ (ppm): 11.01 (s, 1H), 7.78 (s, 1H), 7.31 (s, 1H), 6.95 (s, 1H), 2.87 (s, 3H), 2.63 – 2.52 (m, 2H), 2.42 (s, 3H), 1.70 – 1.46 (m, 2H), 0.90 (t, J = 7.3 Hz, 3H). ^{13}C NMR (75 MHz, DMSO-d_6) δ (ppm): 178.8, 171.9, 166.4, 162.9, 160.0, 155.3, 134.0, 130.9, 120.9, 119.5, 119.3, 106.5, 36.4, 27.2, 26.4, 22.1, 18.9. HRMS (APCI) *m/z* cald. For $C_{17}H_{18}NO_3S$ [M+1]$^+$: 316.1002, found: 316.0985.

7-hydroxy-2,5-dimethyl-3-(4-methylthiazol-2-yl)-4*H*-chromen-4-one (5c): 135.5 mg, 59% yield, Mp 229 – 230ºC. ^{1}H NMR (300 MHz, DMSO-d_6) δ (ppm): 10.72 (s, 1H), 7.27 (s, 1H), 6.73 – 6.65 (m, 2H), 2.83 (s, 3H), 2.70 (s, 3H), 2.41 (s, 3H). ^{13}C NMR (75 MHz, DMSO-d_6) δ (ppm): 180.6, 170.4, 166.6, 162.9, 162.8, 155.3, 147.2, 122.4, 120.6, 120.4, 118.2, 105.5, 27.8, 26.1, 22.2. HRMS (APCI) *m/z* cald. For $C_{15}H_{14}NO_4S$ [M+1]$^+$: 288.0689, found: 288.0685.

2-methyl-3-(4-methylthiazol-2-yl)-4-oxo-6-propyl-4*H*-chromen-7-yl acetate (5d): 44.5 mg, 83% yield, Mp 119 – 120ºC. ^{1}H NMR (300 MHz, $CDCl_3$-*d*) δ (ppm): 8.14 (s, 1H), 7.24 (s, 1H), 7.00 (s, 1H), 2.94 (s, 3H), 2.68 – 2.54 (m, 2H), 2.50 (s, 3H), 2.36 (s, 3H), 1.75 – 1.49 (m, 2H), 0.95 (t, J = 7.3, 3H). ^{13}C NMR (75 MHz, $CDCl_3$-*d*) δ (ppm): 174.9, 168.5, 167.8, 157.9, 153.8, 153.0, 151.1, 132.8, 127.1, 120.4, 116.1, 115.4, 111.2, 31.8, 22.8, 21.6, 20.9, 17.2, 13.8. HRMS (APCI) *m/z* cald. For $C_{19}H_{20}NO_4S$ [M+1]$^+$: 358.1108, found: 358.1107.

6-hydroxy-3-(4-methylthiazol-2-yl)-4*H*-chromen-4-one (8a): 105.7 mg, 51% yield, Mp > 290ºC (Dec); ^{1}H NMR (300 MHz, DMSO-d_6) δ (ppm): 10.21 (s, 1H), 9.20 (s, 1H), 7.64 (d, J = 9.1 Hz, 1H), 7.45 (d, J = 2.9 Hz, 1H), 7.37 – 7.19 (m, 2H), 2.41 (s, 3H). ^{13}C NMR (75 MHz, DMSO-d_6) δ (ppm): 178.8, 161.4,

160.6, 160.5, 156.3, 154.4, 129.1, 128.8, 125.3, 121.5, 121.0, 113.0, 22.0. HRMS (APCI) *m/z* cald. For $C_{13}H_{10}NO_3S$ [M+1]$^+$: 260.0376, found: 260.0379.

6-hydroxy-3-(4-methylthiazol-2-yl)-7-propyl-4*H*-chromen-4-one (8b): 118.1 mg, 49% yield, Mp 261 – 262ºC. ^{1}H NMR (300 MHz, DMSO-d_6) δ (ppm): 10.20 (s, 1H), 9.15 (s, 1H), 7.49 (s, 1H), 7.48 (s, 1H), 7.31 (s, 1H), 2.73 – 2.56 (m, 2H), 2.41 (s, 3H), 1.74 – 1.50 (m, 2H), 0.92 (t, *J* = 7.3 Hz, 3H). ^{13}C NMR (75 MHz, DMSO-d_6) δ (ppm): 178.6, 161.5, 160.2, 158.9, 156.2, 154.4, 143.7, 127.1, 124.3, 121.4, 120.9, 112.3, 37.1, 27.0, 22.0, 19.0. HRMS (APCI) *m/z* cald. For $C_{16}H_{16}NO_3S$ [M+1]$^+$: 302.0845, found: 302.0836.

3-(4-methylthiazol-2-yl)-4-oxo-7-propyl-4*H*-chromen-6-yl acetate (8d): 38.1 mg, 74% yield, Mp 185 – 186ºC. ^{1}H NMR (300 MHz, $CDCl_3$) δ (ppm): 9.07 (s, 1H), 7.96 (s, 1H), 7.44 (s, 1H), 7.01 (s, 1H), 2.70 – 2.53 (m, 2H), 2.50 (s, 3H), 2.37 (s, 3H), 1.85 – 1.54 (m, 2H), 1.00 (t, *J* = 7.3 Hz, 3H). ^{13}C NMR (75 MHz, $CDCl_3$-*d*) δ (ppm): 173.2, 168.3, 155.8, 153.9, 152.8, 150.9, 146.0, 141.9, 121.8, 118.5, 118.1, 117.1, 114.9, 31.7, 21.6, 19.9, 16.2, 13.1. HRMS (APCI) *m/z* cald. For $C_{18}H_{18}NO_4S$ [M+1]+: 344.0951, found: 344.0951.

Spectroscopic Data for the Key Intermediates 3a-e and 7a-c

1-(2,4-dihydroxyphenyl)-2-(4-methylthiazol-2-yl)ethan-1-one (3a): 598 mg, 60% yield; ^{1}H NMR (300 MHz, DMSO-d_6) δ (ppm): 13.85 (s, 1H), 10.16 (s, 1H), 7.46 (d, *J* = 8.8 Hz, 1H), 7.07 (s, 1H), 6.40 (s, 1H), 6.35 (dd, *J* = 8.8, 2.4 Hz, 1H), 6.18 (d, *J* = 2.3 Hz, 1H), 2.32 (s, 3H).

1-(2,4-dihydroxy-5-propylphenyl)-2-(4-methylthiazol-2-yl)ethan-1-one (3b): 583 mg, 50% yield; ^{1}H NMR (300 MHz, DMSO-d_6) δ (ppm): 13.65 (s, 1H), 10.15 (s, 1H), 7.25 (s, 1H), 7.04 (s, 1H), 6.42 (s, 1H), 6.23 (s, 1H), 2.47 – 2.39 (m, 1H), 2.31 (s, 3H), 1.65 – 1.35 (m, 3H), 0.88 (t, *J* = 7.3 Hz, 3H).

1-(2,4-dihydroxy-6-methylphenyl)-2-(4-methylthiazol-2-yl)ethan-1-one (3c): 600 mg, 57% yield; ^{1}H NMR (300 MHz, DMSO-d_6) δ (ppm): 13.79 (s, 1H), 9.97 (s, 1H), 7.08 (s, 1H), 6.44 (s, 1H), 6.22 (s, 1H), 6.08 (d, J = 2.5 Hz, 1H), 2.45 (s, 3H), 2.33 (s, 3H).

1-(2,5-dihydroxyphenyl)-2-(4-methylthiazol-2-yl)ethan-1-one (3d): 224 mg, 45% yield; ^{1}H NMR (300 MHz, DMSO-d_6) δ (ppm): 9.48 (s, 1H), 7.00 – 6.79 (m, 2H), 6.81 – 6.61 (m, 2H), 4.33 (s, 2H), 2.33 (s, 3H).

1-(2,5-dihydroxy-4-propylphenyl)-2-(4-methylthiazol-2-yl)ethan-1-one (3e): 291 mg, 50% yield; ^{1}H NMR (300 MHz, DMSO-d_6) δ (ppm): 9.49 (s, 1H), 6.90 (s, 2H), 6.60 (s, 1H), 4.34 (s, 2H), 2.45 – 2.35 (m, 2H), 2.33 (s, 3H), 1.69 – 1.52 (m, 2H), 0.94 (t, J = 7.3 Hz, 3H).

1-(2,5-dimethoxyphenyl)-2-(4-methylthiazol-2-yl)ethan-1-one (7a): 596 mg, 43% yield; ^{1}H NMR (300 MHz, $CDCl_3$) δ (ppm): 7.37 (dd, J = 3.2, 0.8 Hz, 1H), 7.13 – 6.97 (m, 1H), 6.90 – 6.86 (m, 1H), 6.84 (s, 1H), 4.75 (s, 2H), 3.89 (s, 3H), 3.78 (s, 3H), 2.43 (s, 3H).

1-(4-bromo-2,5-dimethoxyphenyl)-2-(4-methylthiazol-2-yl)ethan-1-one (7b): 890 mg, 50% yield; Keto-Enol tautomerism is present; ^{1}H NMR (300 MHz, $CDCl_3$) (Keto form) δ (ppm): 7.41 (s, 1H), 7.21 (s, 1H), 6.84 (s, 1H), 4.72 (s, 2H), 3.98 – 3.79 (m, 6H), 2.42 (s, 3H). ^{1}H NMR (300 MHz, $CDCl_3$) (Enol form) δ (ppm): 13.70 (s, 1H), 7.41 (s, 1H), 7.21 (s, 1H), 7.14 (s, 1H), 6.84 (s, 1H), 3.93 – 3.80 (m, 6H), 2.42 (s, 3H).

1-(2,5-dimethoxy-4-propylphenyl)-2-(4-methylthiazol-2-yl)ethan-1-one (7c): 639 mg, 80% yield; ^{1}H NMR (300 MHz, $CDCl_3$) δ (ppm): 7.38 (s, 1H), 6.84 (s, 1H), 6.76 (s, 1H), 4.77 (s, 2H), 3.90 (s, 3H), 3.80 (s, 3H), 2.69 – 2.54 (m, 2H), 2.45 (s, 3H), 1.72 – 1.51 (m, 2H), 0.96 (t, J = 7.4 Hz, 3H).

References

[1] L. Boukharta, H. Gutiérrez-de-Terán, J. Åqvist, *PLoS Comput. Biol.* **2014**, *10*, e1003585.

[2] H. Keränen, J. Åqvist, H. Gutiérrez-de-Terán, *Chem. Commun.* **2015**, *51*, 3522–3525.

[3] W. Jespers, G. V. Isaksen, T. A. H. Andberg, S. Vasile, A. van Veen, J. Åqvist, B. O. Brandsdal, H. Gutiérrez-de-Terán, *J. Chem. Theory Comput.* **2019**, acs.jctc.9b00538.

[4] W. Liu, E. Chun, A. A. Thompson, P. Chubukov, F. Xu, V. Katritch, G. W. Han, C. B. Roth, L. H. Heitman, A. P. IJzerman, et al., *Science* **2012**, *337*, 232–6.

[5] H. Gutiérrez-de-Terán, A. Massink, D. Rodríguez, W. Liu, G. W. Han, J. S. Joseph, I. Katritch, L. H. Heitman, L. Xia, A. P. Ijzerman, et al., *Structure* **2013**, *21*, 2175–85.

[6] T. a Halgren, R. B. Murphy, R. A. Friesner, H. S. Beard, L. L. Frye, W. T. Pollard, J. L. Banks, *J. Med. Chem.* **2004**, *47*, 1750–9.

[7] M. Congreve, S. P. Andrews, A. S. Dore, K. Hollenstein, E. Hurrell, C. J. Langmead, J. S. Mason, I. W. Ng, B. Tehan, A. Zhukov, et al., *J. Med. Chem.* **2012**, *55*, 1898–1903.

[8] H. Gutiérrez-de-Terán, X. Bello, D. Rodríguez, *Biochem. Soc. Trans.* **2013**, *41*, 205–12.

[9] D. Rodríguez, H. Gutiérrez-de-Terán, *Proteins* **2012**, *80*, 1919–28.

[10] D. Rodríguez, Á. Piñeiro, H. Gutiérrez-de-Terán, *Biochemistry* **2011**, *50*, 4194–208.

[11] G. A. Kaminski, R. A. Friesner, J. Tirado-Rives, W. L. Jorgensen, *J. Phys. Chem. B* **2001**, *105*, 6474–6487.

[12] W. L. Jorgensen, D. S. Maxwell, J. Tirado-Rives, *J. Am. Chem. Soc.* **1996**, *118*, 11225–11236.

[13] O. Berger, O. Edholm, F. Jähnig, *Biophys. J.* **1997**, *72*, 2002–13.

[14] P. Bauer, A. Barrozo, M. Purg, B. A. Amrein, M. Esguerra, P. B. Wilson, D. T. Major, J. Aqvist, S. C. L. Kamerlin, *SoftwareX* **2018**, DOI 10.1016/j.softx.2017.12.001.

[15] J. Marelius, K. Kolmodin, I. Feierberg, J. Åqvist, J. Aqvist, *J. Mol. Graph. Model.* **1998**, *16*, 213–225.

[16] G. King, A. Warshel, *J. Chem. Phys.* **1989**, *91*, 3647.

[17] F. Lee, A. Warshel, *J. Chem. Phys.* **1992**.

[18] J.-P. J. Ryckaert, G. Ciccotti, H. J. . H. Berendsen, *J. Comput. Phys.* **1977**, *23*, 327–341.

[19] W. Jespers, M. Esguerra, J. Åqvist, H. Gutiérrez-de-Terán, *J. Cheminform.* **2019**, *11*, 26.

[20] M. J. Robertson, J. Tirado-Rives, W. L. Jorgensen, *J. Chem. Theory Comput.* **2015**, *11*, 3499–3509.

[21] J. L. Banks, H. S. Beard, Y. Cao, A. E. Cho, W. Damm, R. Farid, A. K. Felts, T. A. Halgren, D. T. Mainz, J. R. Maple, et al., *J. Comput. Chem.* **2005**, *26*, 1752–1780.

[22] C. H. Bennett, *J. Comput. Phys.* **1976**, *22*, 245–268.

[23] W. Kabsch, *Acta Crystallogr. Sect. D Biol. Crystallogr.* **2010**, *66*, 133–144.

[24] P. Evans, in *Acta Crystallogr. Sect. D Biol. Crystallogr.*, **2006**, pp. 72–82.

[25] M. D. Winn, C. C. Ballard, K. D. Cowtan, E. J. Dodson, P. Emsley, P. R. Evans, R. M. Keegan, E. B. Krissinel, A. G. W. Leslie, A. McCoy, et al., *Acta Crystallogr. Sect. D Biol. Crystallogr.* **2011**, *67*, 235–242.

[26] P. R. Evans, G. N. Murshudov, *Acta Crystallogr. Sect. D Biol. Crystallogr.* **2013**, *69*, 1204–1214.

[27] A. J. McCoy, R. W. Grosse-Kunstleve, P. D. Adams, M. D. Winn, L. C. Storoni, R. J. Read, *J. Appl. Crystallogr.* **2007**, *40*, 658–674.

[28] C. Vonrhein, I. J. Tickle, C. Flensburg, P. Keller, W. Paciorek, A. Sharff, G. Bricogne, *Acta Crystallogr. Sect. A Found. Adv.* **2018**, *74*, a360–a360.

[29] P. V. Afonine, R. W. Grosse-Kunstleve, N. Echols, J. J. Headd, N. W. Moriarty, M. Mustyakimov, T. C. Terwilliger, A. Urzhumtsev, P. H. Zwart, P. D. Adams, *Acta Crystallogr. Sect. D Biol. Crystallogr.* **2012**, *68*, 352–367.

[30] P. Rucktooa, R. K. Y. Cheng, E. Segala, T. Geng, J. C. Errey, G. A. Brown, R. M. Cooke, F. H. Marshall, A. S. Doré, *Sci. Rep.* **2018**, *8*, 41.

[31] P. Emsley, B. Lohkamp, W. G. Scott, K. Cowtan, *Acta Crystallogr. Sect. D Biol. Crystallogr.* **2010**, *66*, 486–501.

[32] G. Bricogne, E. Blanc, M. Brandl, C. Flensburg, P. Keller, W. Paciorek, P. Rovers, A. Sharff, O. Smart., C. Vonrhein, T.O. Womack. BUSTER version 2.11.7. Global Phasing Ltd.: Cambridge, United Kingdom: (2017).

[33] The PyMOL Molecular Graphics System, Version 1.4 Schrödinger, LLC.

[34] A. Zhukov, S. P. Andrews, J. C. Errey, N. Robertson, B. Tehan, J. S. Mason, F. H. Marshall, M. Weir, M. Congreve, **2011**, 4312–4323.
